# Wet Season Upwelling and Dry Season Chlorophyll-a Describe Interannual Growth Rates of *Porites* in Southern China

**DOI:** 10.1371/journal.pone.0099088

**Published:** 2014-06-05

**Authors:** Teng Teng Yang, Nathalie Fairbank Goodkin

**Affiliations:** 1 Department of Earth Sciences, The University of Hong Kong, Hong Kong; 2 Swire Institute of Marine Science, The University of Hong Kong, Hong Kong; Seagrass Ecosystem Research Group, Swansea University, United Kingdom

## Abstract

Southern China hosts coral communities in marginal environments that are characterized by low linear extension rates, low coral cover and/or no reef formation, thus providing natural laboratories to study coral communities with below average growth rates. Here we compare the annual linear extension rates over 10 years (range 1.2 to 11.4 mm yr^−1^) of six *Porites* sp. coral cores collected from Hong Kong with monthly hydrographic data from the Hong Kong Environmental Protection Department. At all sites, low-density, dry season extension were more variable than high-density, wet season extension and on average, was lower at two of the three sites. We applied multi-variate linear regressions that revealed high-density, wet season band extension to inversely correlate most significantly to temperature (r = −0.39, p<0.01). In contrast, low-density, dry season band extension was more variable and correlated most significantly with dry season chlorophyll-a (Chl-a) (r = 0.64, p<0.001). Additionally, we find that corals at the site with highest dry season Chl-a have the highest dry season extension lengths. Our findings indicate that relative mixing of fresh and salt water in the wet season and primary productivity in the dry season, and their influences on aragonite saturation, are likely to impact interannual coral extension variability in marginal environments.

## Introduction

Marginal coral communities, classified by low coral cover and/or no reef formation, generally have multiple limiting environmental conditions, including low seawater temperatures, low pH, high nutrient concentrations, low light, and high turbidity [1]–[7]. However, marginal conditions do not always negatively impact species diversity, as healthy and marginal communities can exhibit similar levels of diversity [3], [5], [8]. Seasonally variable and generally low temperatures are a common factor in marginal reefs [7], and coral extension and calcification rates have been shown to be positively correlated with temperature, with linearity diminishing at both extreme high and low temperatures [9]–[14]. Marginal communities are also found in environments with variable or low salinities [12], [15], [16] or areas where high coastal runoff leads to nutrient-enriched and usually, oxygen-depleted waters [17]–[19].

Most studies of marginal corals have quantified the relationships between coral growth and only one or two environmental parameters, such as temperature, salinity, turbidity, or pH [10], [13], [14], [20]–[22]. Some studies have investigated the impact of multiple environmental conditions on growth rates [23]–[25]. However, there are relatively few studies that examine how the combination of different environmental conditions in one region impacts the growth of individual colonies [26]. Therefore, our knowledge of how environmental conditions combine to impact coral growth is limited, as each species, and the same species in different regions, exhibit varying growth responses to environmental conditions [11].

**Figure 1:**
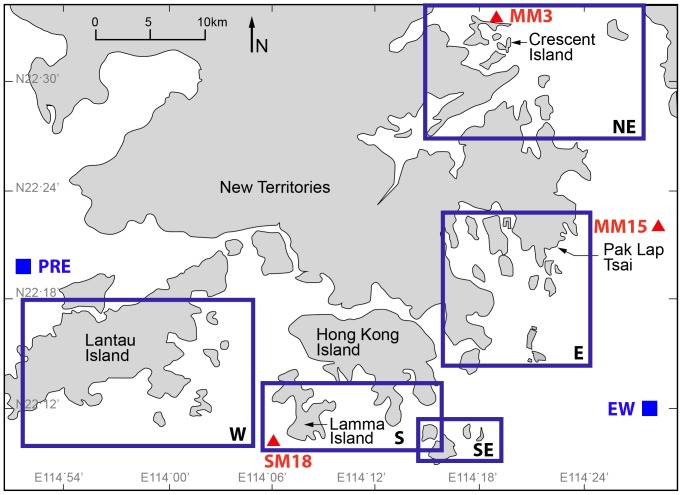
Map of the Hong Kong marine environment, including the Pearl River Estuary (PRE) to the west. Boxes (NE, E, SE, S and W) are regions of similar coral communities previously identified [8], [12]. Coral coring sites (Lamma Island, Pak Lap Tsai and Crescent Island) from the three regions with long-lived coral species are marked (red circles). Environmental Protection Department (EPD) hydrographic sites used in this study are identified (red triangles). The Pearl River Estuary (PRE) and eastern open ocean waters (EW) end member sites are shown (blue squares) [39].

The coastal waters of Hong Kong provide a natural environment to study the impacts of varying physical conditions on coral growth. Corals have been found in Hong Kong for centuries; however, no reef formation is recorded and coral growth rates are low [12], [27]–[30]. Over the past decade, the Hong Kong Environmental Protection Department (EPD) has maintained more than 70 marine water quality hydrographic stations, which provide a wealth of information about marine conditions. Here, we study the relationship between physical conditions during the wet and dry seasons and interannual growth rates of six *Porites* sp. from three different regions within Hong Kong (Figure 1).

### Study Site: Hong Kong

Hong Kong (22°23′N 114°10′E, Figure 1), in southern China, is located on the eastern end of the Pearl River Estuary, with estuarine waters in the west and southwest and open-ocean conditions to the east. In addition to the environmental gradient, proximity to the Asian continent amplifies seasonal climatic variability. Hong Kong waters exhibit subtropical temperature ranging from 13.9°C in the winter dry season to 31.5°C in the summer wet season [12], [31]. Hong Kong waters experience a large range of nutrient variability, with total inorganic nitrogen ranging from 0.01 to 1.41 mg L^−1^ and chlorophyll-a (Chl-a) seasonally ranging from 0.20 to 35 µg L^−1^ [31], [32]. Southwesterly winds in summer cause localized upwelling along Hong Kong’s coast, whereas northeasterly winter winds lead to localized downwelling [32]. The ocean circulation around Hong Kong maintains nutrient limitation outside of the estuary throughout the year [33]. Spatially, salinity and pH range from 11.3 to 35.2 psu and from 7.4 to >8.5, respectively [31]. The April to September wet season delivers fresh, turbid and nutrient-rich water from the Pearl River Estuary into western Hong Kong waters where no hard corals are found [8], [12]. Turbidity (0.3–52.0 normal turbidity units (NTU)) and dissolved oxygen (DO) (1.5–11.8 mg L^−1^) [31] are outside the range typically found on large coral reef formations [3], [7], [12], [34].

Modern coral colonies in Hong Kong are found scattered on outlying islands in shallow coastal waters, with no evidence of a coral reef framework now or in the past [8], [12], [27], [35]. Coral cover and biodiversity increase toward the eastern and northeastern waters, reaching greater than 50% coral cover, and 36% of all species found in Hong Kong are found here [8], [12]. Soft corals dominate the western region, and long-lived corals (>50 years) are only found in the previously defined south, east, and northeast regions. Century-old *Porites* in Hong Kong grow <4 mm yr^−1^ [12], half the rate for the same species in non-marginal environments [9], [13], [20], [25]. In this study, we collected six *Porites* coral cores from Hong Kong waters from three sites - Lamma Island (22°11.336′N; 114°7.942′E, depth 7.3 and 7.9 m), Pak Lap Tsai (22°21.141′N; 114°22.152′E, depth 9.0 and 9.5 m) and Crescent Island (22°31.848′N; 114°18.897′E, depth 2.2 and 3.4 m) (Figure 1). The three study sites are geographically distinct and represent the three different environments in Hong Kong hosting long-lived corals. Lamma Island, located to the southwest, is nearest to the Pearl River Estuary with an average water depth of 7 m. Pak Lap Tsai is located in the open-ocean waters of the southeast, where average water depths exceed 10 m. Crescent Island is located in northern Hong Kong, in proximity to mainland China. This island is partially sheltered by the Shenzhen coast, and corals are generally found in shallow (∼3 m) semi-enclosed basins.

## Methods

### a) Coral Coring and Extension Rate Analysis

A pneumatic hand-held drill connected to a modified, high-pressure air compressor was used to collect cores of 5 cm in diameter and 30–80 cm in length. Permission to collect corals was granted by the Agriculture, Fisheries, and Conservation Department of the Government of Hong Kong, Special Administrative Region of China. Core holes were filled with a mixture of silt and coarse sand and plugged with cement. Samples HK005 and HK006 were collected whole as they were too small to drill. Samples were cleaned with deionised water and dried under the sun. The cores were labelled and cut longitudinally along the maximum growth axis. Slabs of ∼10 mm thickness were obtained using a diamond-coated trim saw. The slabs were rinsed with deionised water 10 times, submerged in an ultrasonic bath three times for 10 minutes, and then dried at 60°C for 24 hours [36].

**Figure 2:**
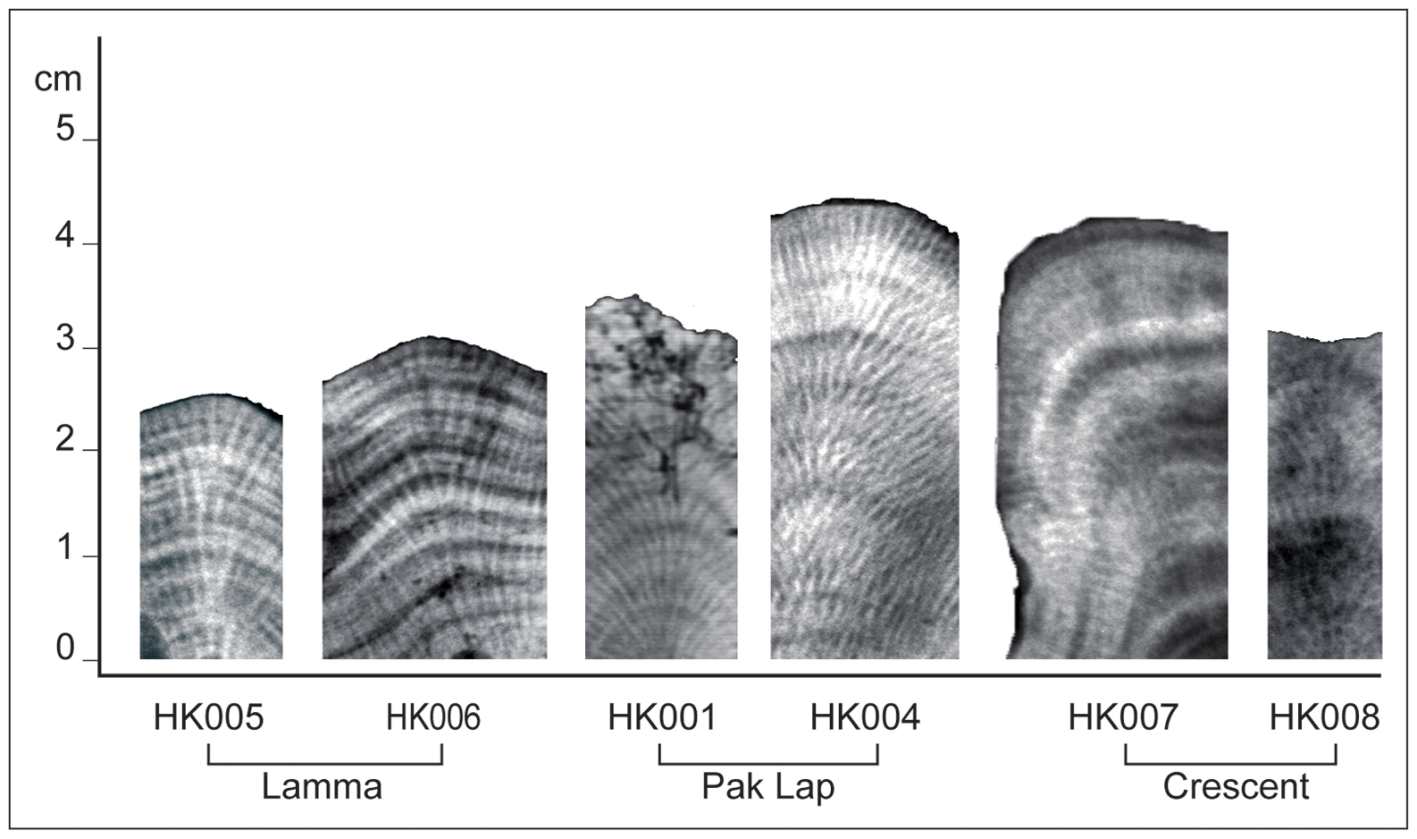
Coral X-radiograph negatives from the three regions: Lamma Island (HK005, HK006), Pak Lap Tsai (HK001, HK004), and Crescent Island (HK007, HK008).

Each coral slab was X-radiographed at the Biomedical Imaging Center in Hong Kong with the following settings: film focus distance = 100 cm; distance between machine and coral = 110 cm, exposure = 0.16 sec, voltage = 48 kV, current = 20 mA. Linear extension (growth) was measured along the primary, axial growth axis where the polyps were perpendicular to the line of slicing [37]. Linear extension was measured from digital images of the X-radiograph negatives, so light bands on the film represent high-density bands and dark bands represent low-density bands. Seasonal high- (light) and low- (dark) density growth was calculated by measuring the height of each band (Figure 2). Digital images of each coral slab were magnified up to 300% to distinguish the borders between each high- and low-density band, and measurements were made digitally using guides and rulers. Sample HK001 had very low extension rates and significant bioerosion at the surface, so extension was quantifiable only for 2000–2002.

### b) Environmental Data

Since 2000, the EPD has collected monthly hydrographic data from >70 sites in and around Hong Kong (http://www.epd.gov.hk/epd/english/environmentinhk/water/marine_quality/mwq_home.html). EPD data from 2000–2010 were extracted for three sites: SM18 (21 m depth, Lamma Island), MM15 (24 m depth, Pak Lap Tsai), MM3 (16 m depth, Crescent Island) (Figure 1). EPD data is collected at three depths - the surface (1 m below surface), the mid-depth (half-way between the surface and the bottom) and the bottom (1 m above the bottom). Mid-depth parameters were used in this study. Surface depths were not used as Hong Kong experiences strong seasonal thermal stratification [32], and corals are not generally found within 1 m of the surface. Most corals are in shallower water columns than the EPD sites, thus mid-depth is most applicable.

The seven environmental variables temperature (T), salinity (S), dissolved oxygen (DO), turbidity, chlorophyll a (Chl-a), total inorganic nitrogen (TIN) and pH were used, as previous studies showed that these are most relevant for coral growth [2], [7], [12], [24], [38]. Neither aragonite saturation nor the parameters needed to calculate aragonite saturation are available from the EPD data. However, previous studies provide an indication of the relationship between salinity, dissolved inorganic carbon, primary production, and aragonite saturation, which will be discussed later [33], [39], [40]. The environmental data was missing for October 2001 and 2008 at all sites and January and February 2000 at MM15. Missing months were calculated by linear interpolation. Hong Kong experiences two clear seasons – wet (April-September) and dry (October-March). Data from each site was averaged into wet and dry seasons for comparison to the extension data.

### c) Statistical Analysis

Physical parameters from EPD stations at Lamma Island-SM18, Pak Lap Tsai-MM15, and Crescent Island-MM3 from 2000–2009 were used to analyze relationships between seasonal extension rates and environmental conditions for the 10 year period. High- and low-density band lengths were correlated to all seven environmental conditions from both the wet and dry seasons to identify parameters independently correlating with the band length. Pearson correlations were used for both the complete data set and the data set excluded years of exceptional growth (>3 times a quartile) [41]. Physical parameter outliers were then identified (>3 times a quartile) and the correlations were checked for significance excluding these outliers. Conditions that correlate independently with the band length in all scenarios with a statistical significance of p<0.05 were then regressed with the band length as a group, using a step-wise multi-variate type-1 regression in SPSS [42] to identify the most significant parameters. If parameters from two seasons both correlated with one season of banding, a Durbin Watson (DW) test was conducted to evaluate co-linearity through time.

### d) Evaluation of Changes to Ω

The saturation state of aragonite (Ω) between the Pearl River Estuary and open ocean water in Hong Kong was estimated by using previously published *in situ* measurements of T, S, and dissolved inorganic carbon (DIC) with pH measurements from coral sites to investigate relative changes to Ω [39]. These two sites represent end members of the environmental conditions experienced by corals in Hong Kong. Ω before and after an algal bloom was calculated using measurements of T, S, DIC, alkalinity, and pH reported by Dai *et al.* (2008) [40]. DIC is assumed to be equal to the concentration of carbonate, CO_3_^ = ^, and bicarbonate, HCO_3_^−^, neglecting the very minor contribution by H_2_CO_3_.
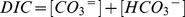
(1)


K_2_, the dissociation constant of bicarbonate to carbonate was calculated as a function of T and S according to Emerson and Hedges (2008) [43]:
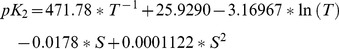
(2)where T is in degrees kelvin, S is in psu, and K_2_ is in mol kg^−1^. K_2_ is equal to:



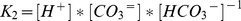
(3)Substituting equation 1 into equation 2 allows for the calculation of the concentration of carbonate. The concentration of calcium in seawater can be calculated as a function of salinity following Libes (1992) [44]:
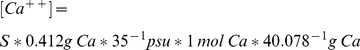
(4)


The solubility product of aragonite, K_sp_, is calculated as a function of T and S according to Mucci (1983) [45] and reprinted in Zeebe and Wolf-Gladrow (2001) [46]:
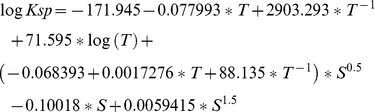
(5)where, T is in degrees kelvin, S is in psu, and K_sp_ is in mol^2^ kg^−2^.

Finally, Ω is equal to:
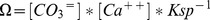
(6)


## Results

**Figure 3:**
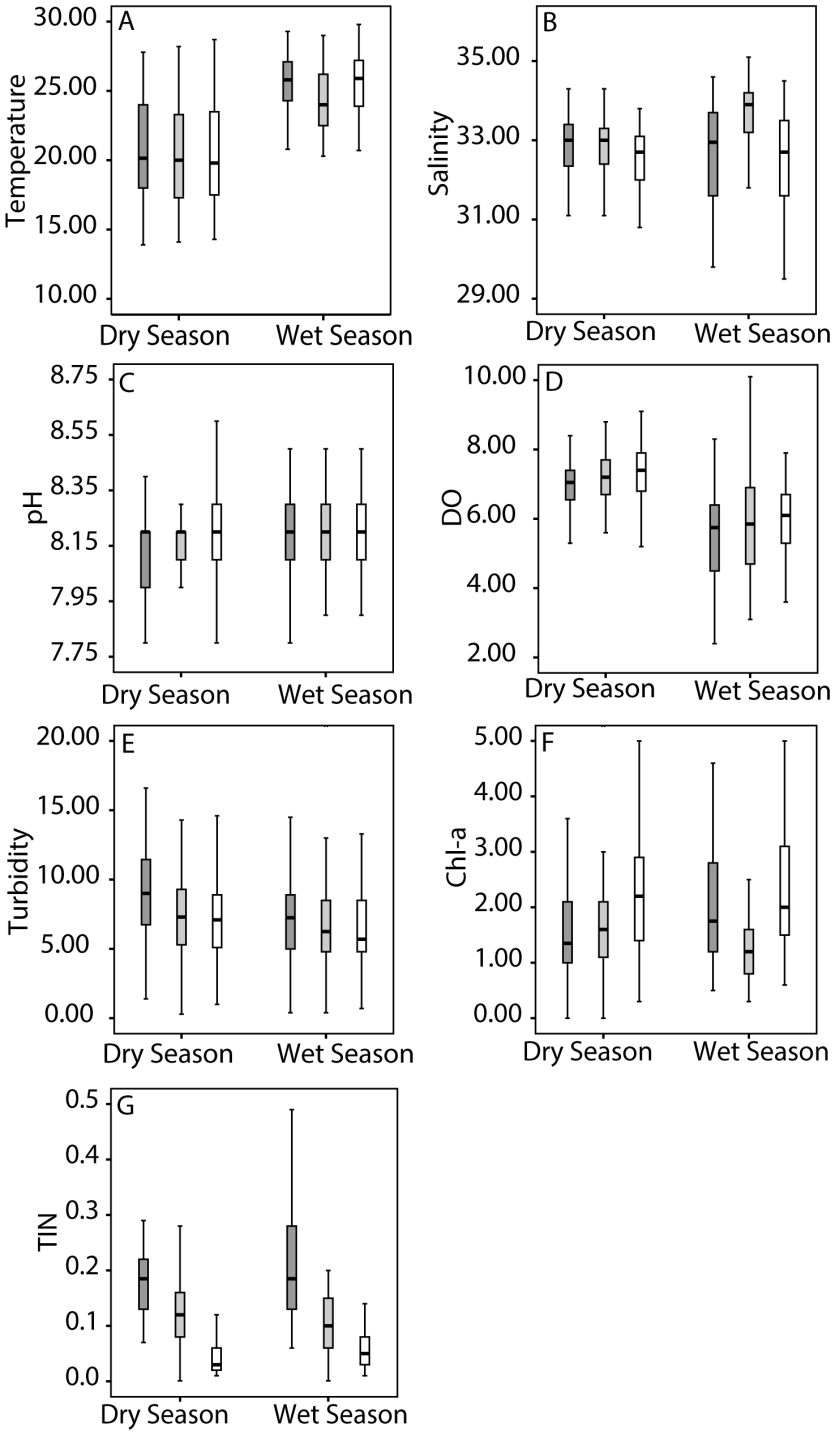
Monthly hydrographic data divided into the wet season (April-September) and the dry season (October-March) plotted to show the median (bold mid-line), the first and third quartiles (box), and 1.5 times each quartile (black lines) for Lamma Island (dark shading), Pak Lap Tsai (light shading) and Crescent Island (no shading). Data is shown for a) temperature (deg C), b) salinity (psu), c) *in situ* pH, d) dissolved oxygen (mg L^−1^), e) turbidity (NTU), f) chlorophyll-a (µg L^−1^), and g) total inorganic nitrogen (mg L^−1^).

For T and DO, raw monthly data averaged into wet and dry seasons show offsets in the mean values between seasons at all sites (Figure 3). The pH means exhibited little variation between seasons at all sites. In contrast, TIN showed a decreasing trend from the southeast to northeast sites in both seasons.

**Figure 4:**
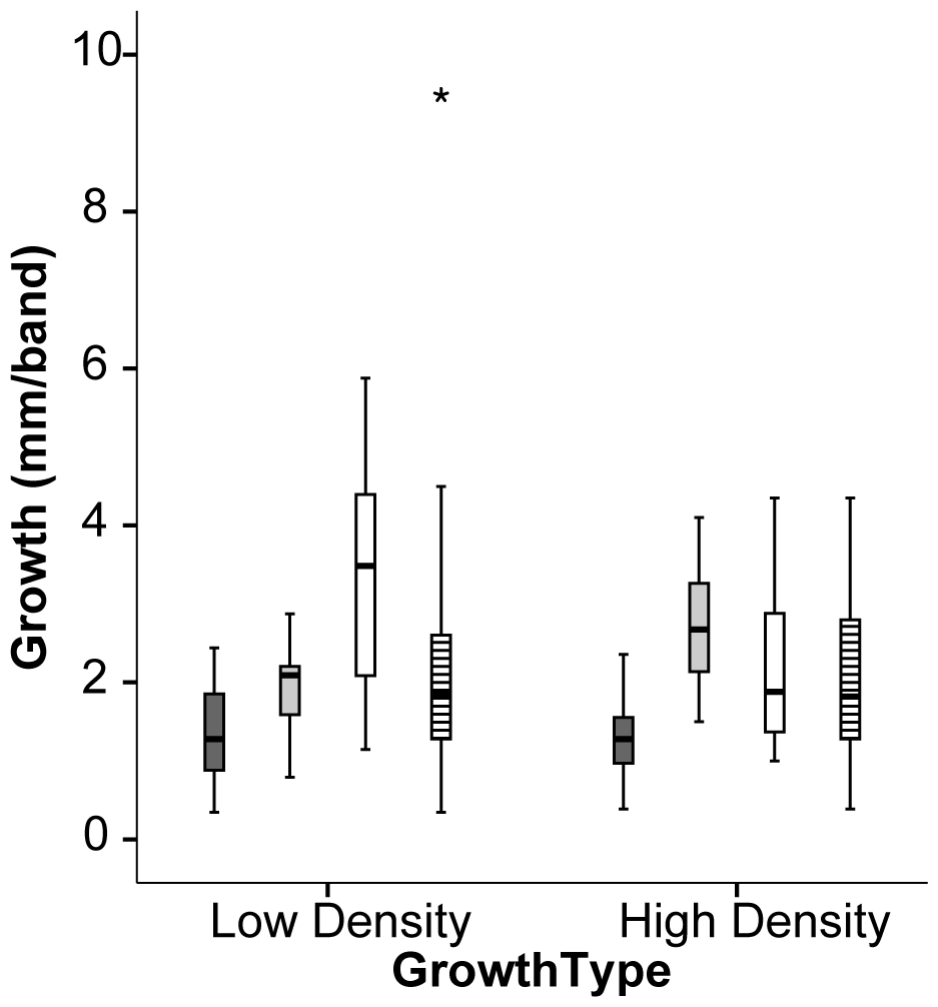
Coral extension length (mm band^−1^) plotted to show the median (bold mid-line), the first and third quartiles (box), and 1.5 times each quartile (black lines) for low-density (dark) and high-density (light) banding. The bandings are described for Lamma Island (dark shading), Pak Lap Tsai (light shading), Crescent Island (no shading), and all sites (striped). Outlier (star) for all three sites was defined as >3 times each quartile.

From 2000–2009, the six corals had annual extensions ranging from 1.2 mm yr^−1^ to 11.4 mm yr^−1^, with an average of 4.4 (±2.2) mm yr^−1^ (Table 1, Figure 4). *Porites* found near Lamma Island (HK005, HK006), showed the lowest and least variable growth. HK005 exhibited the lowest average annual extension (2.2±0.6 mm yr^−1^) among all cores while HK006 from the same site averaged 3.7±1.7 mm yr^−1^. The Pak Lap Tsai cores (HK001, HK004) showed average growth rates of 3.7±1.2 and 4.8±1.0 mm yr^−1^, respectively. Linear extension was greatest in the Crescent Island cores (avg. of 4.9±2.1 and 7.6±2.3 mm yr^−1^), though lower than a previously published rate of 10 mm yr^−1^ from the northeastern region [47].

**Table 1:** Average annual linear extension rate (mm yr^−1^), standard deviation (S.D.), range of annual extension rates (minimum-maximum), period of analysis and water depth (m) for six *Porites* cores.

	Lamma Island		Pak Lap Tsai		Crescent Island	
	HK005	HK006	HK001	HK004	HK007	HK008
Average (mm yr^−1^)	2.2	3.7	3.7	4.8	4.9	7.6
S.D.	0.6	1.7	1.2	1.0	2.1	2.3
Range (min–max)	1.3–3.4	1.2–5.9	2.3–4.9	3.6–6.0	2.5–8.5	4.7–11.4
Duration studied (years)	2000–2009	2002–2009	2000–2007	2000–2009	2000–2009	2004–2009
Depth (m)	7.3	7.9	9.0	9.5	2.2	3.4

Linear extension of the low-density, dry season bands (dark) was significantly more variable than that of the high-density, wet season bands (light) (Variance = 2.7 and 1.3 mm^2^ respectively, F-test p = 0.01). The Lamma Island and Pak Lap Tsai corals generally exhibited greater extension during high-density banding (avg. 1.5 and 2.7 mm per band, respectively) than low-density banding (avg. 1.4 and 1.8 mm per band, respectively) (t-test = 2.77, p = 0.01). In contrast, Crescent Island corals exhibited low-density growth that tended to be higher than high-density growth (avg. 3.6 and 2.3 mm per band, respectively) (t-test = −2.27, p = 0.04). Crescent Island showed greater low-density variability than the other two sites (Variance = 4.2 and 0.5 mm^2^ respectively, F-test p<<0.0001), although Crescent Island’s high-density variability was not significantly different from that of Lamma Island and Pak Lap Tsai (Variance = 1.9 and 1.1 mm^2^, respectively, F-test p = 0.11).

High-density, light band extension correlated significantly (p<0.05) with wet season T (inverse) and S (Table 2). Neither high-density banding, wet season T, nor S had any outliers with absolute values greater than three times the upper or lower quartile. No additional wet or dry season parameters were significantly correlated with high-density banding. A step-wise linear regression of all high-density bands with the two significantly correlated parameters above identified wet season T alone as the most significant predictor. These two wet season physical parameters were significantly correlated (slope = −0.73°C psu^−1^, r = −0.58, p<0.001, n = 47, Table 2).

**Table 2:** Pearson correlation statistics reported for high-density (light) bands compared to wet season (WS) temperature (°C) and salinity (psu) for all points.

		All Points	
		WS Salinity	WS Temperature
**High-density Band**	Pearson Correlation	0.31	−0.39
	Sig. (2-tailed)	0.034	0.006
	N	47	47
**WS Salinity**	Pearson Correlation		−0.58
	Sig. (2-tailed)		0.000
	N		47

Statistics include the Pearson correlation (r-value), Significance (2-tailed, p-value) and the sample size (N).

Low-density, dark band extension was significantly correlated (p<0.05) with wet season TIN (inverse) and dry season DO, Chl-a, and TIN (inverse) both with and without the growth outlier (Table 3). Dry season Chl-a also had two data points more than three times the upper quartile for the two Crescent Island corals; however, removal of these outliers did not render the relationship insignificant (r = 0.34, p<0.05, n = 43). A step-wise linear regression of all significant parameters identified dry season Chl-a alone as the best model to describe low-density banding. Wet season TIN was significantly correlated and co-linear with dry season DO (r = −0.36, p<0.05, n = 47 and DW = 0.636) and dry season TIN (r = 0.88, p<<0.01, n = 47 and DW = 1.432). Dry season DO was also significantly correlated with dry season Chl-a (r = 0.67, p<<0.01, n = 47) (Table 3).

**Table 3:** Pearson correlation statistics for low-density (dark) bands compared to wet season (WS) total inorganic nitrogen (TIN), dry season (DS) dissolved oxygen (DO), chlorophyll-a (Chl-a), and TIN and for the environmental parameters compared to one another.

		All Points				Removed Growth Outlier			
		WS TIN	DS DO	DS Chl-a	DS TIN	WS TIN	DS DO	DS Chl-a	DS TIN
**Low-density Band**	Pearson Correlation	−0.37	0.54	0.64	−0.41	−0.37	0.39	0.48	−0.47
	Sig. (2-tailed)	0.010	0.000	0.000	0.004	0.011	0.008	0.001	0.001
	N	47	47	47	47	46	46	46	46
**WS TIN**	Pearson Correlation		−0.36	−0.27	0.88				
	Sig. (2-tailed)		0.013	0.063	0.000				
	N		47	47	47				
**DS DO**	Pearson Correlation			0.67	−0.19				
	Sig. (2-tailed)			0.000	0.195				
	N			47	47				
**DS Chl-a**	Pearson Correlation				−0.275				
	Sig. (2-tailed)				0.061				
	N				47				

Statistics are shown for all data points and with the growth outlier removed. Statistics include the Pearson correlation (r-value), Significance (2-tailed, p-value) and the sample size (N).

Aragonite saturation (Ω) in the Pearl River Estuary and eastern open ocean waters was 3.3 and 4.1, respectively (Figure 1, Table 4). Furthermore, an algal bloom moving from the Pearl River Estuary to coastal waters off Hong Kong [40] likely raised Ω from 4.5 pre-bloom to 6.5 post-bloom.

**Table 4:** Estimated values of aragonite saturation (Ω) using equations 1–6 and data from Yuan *et al*. (2011) [39] for wet season Pearl River Estuary and Eastern Open Ocean Waters for both surface and bottom.

	Surface Water	Bottom Water
Pearl River Estuary	3.3±0.5	4.1±0.6
Eastern Open Ocean Waters	3.9±0.6	4.1±0.6
Open Ocean Waters		
Pre-Bloom	4.5	
Post-Bloom	6.5	

The mean wet season pH of 8.18 was used with top and bottom quartile pHs to estimate the error. Data from Dai *et al*. (2008) [40] was used for pre- and post-bloom calculations at ∼10 m depth including pH.

## Discussion

Our coral extension rate results must take into account local variability, measurement errors and the possibility of confounding influences. First of all, the exact timing of growth banding in Hong Kong is unknown and changes in timing between colonies and between years would be expected [48]. In addition, growth banding is more likely to follow a sine wave approximation of density rather than the bimodal method used here to approximate the seasons [49]. Finally, error in our calculated extension rates likely exists from the two-dimensional nature of x-radiographs and the inability to differentiate between extension and infilling of carbonate materials [50]. These caveats aside, it is highly likely, given the >10°C temperature variability, that annual banding generally follows the large seasonal temperature shifts as found in other sub-tropical locations where high-density (light) banding occurs in warm temperatures and vice versa [51], [52]. While there is no perfect method for estimating growth rates from coral skeletons, the methods employed here follow established protocols and give a good measure of interannual variability.

Our assumption that coral banding was likely to follow seasonal temperature shifts appears to be sound, as the high-density (light) banding correlated only with wet season (warm T) parameters and the low-density (dark) banding correlated primarily with dry season (cold T) parameters, but also with wet season TIN (Table 2 and 3). Wet season TIN was more strongly correlated with another significant parameter, dry season TIN than with low-(dark) density banding. This indicates that the correlation to dark banding may arise from the co-linearity of wet season and dry season TIN and wet season TIN and dry season DO. Best-fit models for both high-density and low-density banding were seasonally independent from one another as per our original hypothesis.

Coral extension is commonly believed to be highly positively correlated with temperature [9]. Previously, low temperatures were found to explain the significantly depressed average extension rates in Hong Kong [12]. Although average growth is likely driven by low average T, interannual growth variability in the wet season is inversely related to temperature and appears to depend on the relative proportion of seawater and freshwater mixing, while interannual growth in the dry season is related to surface water algal blooms.

High-density growth is most significantly described by its inverse relationship with wet season temperature. Wet season temperature in Hong Kong can exceed 30°C, but averages ∼25°C. On average, wet season temperature was lower than for typical non-marginal reefs, and should not regularly lead to thermal stress [7]. The significant inverse correlation between wet season T and S (slope = −0.73°C psu^−1^, r = −0.58, p<0.001, n = 47, Table 2) indicates that the wet season T is a proxy of fresh- versus saltwater mixing. In the wet season, Hong Kong experiences upwelling, causing cold, salty water to mix with fresh, warm water from both river run-off and precipitation [33]. As upwelling rates would increase, temperature decreases and salinity increases. In this scenario, the thermal sensitivity of growth is less than the haline sensitivity, given the magnitude of ΔT versus ΔS during wet season months.

In addition to T and S, aragonite saturation (Ω) is likely to play a role in coral extension. While we have no direct measure of Ω, the literature provides an understanding of changes to Ω under varying conditions. Using data from Yuan *et al*. (2011) [39], Ω increases from 3.3 to 4.1 from the Pearl River Estuary to eastern open ocean waters. The corals are likely to experience a smaller change than found between these two points, as they are not likely to experience the pure Pearl River Estuary end member. However, it has been shown that below an Ω of ∼5, coral calcification rates will decrease at a rate of roughly 20% per Ω [53]. In the wet season, the root mean square of the difference between light, high-density band extension and the average extension varies relative to the average extension between 20–60%, equal to or larger than the effect due to just to a shift of 1 in Ω. Changes to saturation state due to mixing of ocean and estuary water during the wet season likely contributes to interannual extension variability along with haline stress.

In contrast to the wet season, when physical circulation appears to play a role in growth variability, dry season, low-density growth is most significantly described by variability in Chl-a (Table 4). Chl-a, an indicator of phytoplankton growth, leads to increased extension likely by increasing both food availability for heterotrophy and signaling improved environmental conditions for photosynthesis of both plankton and coral symbionts [54], [55]. Simultaneously, DO increases in the dry season likely because of lower temperature increases oxygen solubility, because of increased primary production, and the cessation of wet season upwelling supplying low DO waters to the surface [33], [39]. Rates of skeletogenesis have previously been related to symbiont photosynthesis, coral feeding and organismic energy resources [56], [57]. The Crescent Island site has the highest dry season Chl-a median and the highest dry season extension (Figure 3 and 4), indicating that during the cold, dry season energy supply from either photosynthesis or feeding on algae is critical to extension variability.

Algal blooms indicated by Chl-a will also likely have an impact on Ω. The detailed study of an algal bloom showed that the bloom decreased DIC and alkalinity, and increased pH [40]. Using equations 1–6, we calculated that under these conditions Ω would increase by 2 (Table 4), leading to for a roughly 40% increase in coral calcification rates [53]. Dry season extension rates in Crescent Island, vary 40% above and below the mean indicating that changes to aragonite saturation may explain changes to extension rates correlating with Chl-a.

In conclusion, corals living in marginal environments are already experiencing significant limits to growth. Our study indicates that in Hong Kong, these limits are due to relative estuarine influence in the wet season, and primary production or variable Ω in the dry season.
